# Phase-dependent reorganization of circulating miRNA networks following short maximal exercise

**DOI:** 10.3389/fphys.2026.1852791

**Published:** 2026-06-03

**Authors:** Jana Jaklová Dytrtová, Michal Jakl, Ondřej Morávek, Denisa Smělá, Zuzana Bílková, Tomáš Navrátil, Farwa Baber, Jan Maleček, Lucie Korecká

**Affiliations:** 1 Sport Sciences–Biomedical Department, Faculty of Physical Education and Sport, Charles University, Prague, Czechia; 2 Department of Biological and Biochemical Sciences, Faculty of Chemical Technology, University of Pardubice, Pardubice, Czechia; 3 Department of Electrochemistry at the Nanoscale, J. Heyrovský Institute of Physical Chemistry of the Czech Academy of Sciences, Prague, Czechia; 4 Department of Military Physical Education, Faculty of Physical Education and Sport, Charles University, Prague, Czechia

**Keywords:** biomarker coordination, exercise-induced stress, internal load, microRNA, recovery, tissue oxygen saturation

## Abstract

**Background:**

Circulating microRNAs (miRNAs) are proposed biomarkers of exercise-induced stress and adaptation, yet acute responses are heterogeneous across studies. This suggests that miRNA regulation may depend on the temporal phase of the exercise response and on internal physiological stress, rather than on external workload alone.

**Objective:**

To characterize phase-specific circulating miRNA responses to short maximal-effort exercise and examine their associations with internal physiological stress, recovery dynamics, and performance.

**Methods:**

Eleven healthy young men performed a short maximal-effort cycling bout. Serum miR-103, miR-122, miR-144, and miR-486 were measured before exercise, immediately post-exercise, and 1 h post-exercise. Salivary cortisol and muscle tissue oxygen saturation (StO_2_) were assessed as markers of endocrine and internal physiological stress. Responses were analyzed as log_2_-transformed fold changes across acute (after/before), recovery vs baseline (rest/before), and active recovery (rest/after) phases using non-parametric statistics and Spearman correlations.

**Results:**

No uniform exercise-induced change in individual miRNAs was observed. Instead, responses were phase-specific: the acute phase showed coordinated behavior between miR-122 and miR-486 and alignment of molecular responses with exercise-induced tissue deoxygenation (ΔStO_2_), whereas associations with performance were not evident. At 1 h post-exercise, miRNA levels largely returned toward baseline while salivary cortisol remained elevated. During active recovery, a strong coupling emerged between miR-103 and miR-144, consistent with coordinated metabolic-oxidative regulation.

**Conclusion:**

Short maximal-effort exercise induces a phase-dependent reorganization of circulating miRNA coordination rather than a single uniform miRNA signature. Circulating miRNA dynamics appear to reflect internal physiological stress and early recovery processes more closely than external workload, supporting a phase-specific, network-based interpretation of miRNA regulation in exercise physiology.

## 1 Introduction

MicroRNAs (miRNAs, miRs) are short, single-stranded RNA molecules (21–28 nucleotides) that post-transcriptionally silence or fine-tune target mRNAs, enabling rapid and reversible regulation of metabolic control, immune function, and cellular stress adaptation. Because they can be detected in circulation, miRNAs have gained attention as potential biomarkers of dynamic physiological changes, including those induced by physical exercise. Exercise responses are traditionally assessed using established biochemical and hormonal markers such as cortisol, a key indicator of hypothalamic–pituitary–adrenal axis activation. While informative, cortisol reflects an integrated endocrine output whose apparent temporal resolution depends strongly on sampling timing and exercise intensity. Circulating miRNA profiles may complement endocrine markers by capturing post-transcriptional regulation and coordinated, phase-specific molecular responses across tissues, rather than replacing conventional stress markers.

Regular physical activity is a fundamental determinant of health, whereas insufficient activity is associated with muscle atrophy (Grondard et al., 2005), obesity (Elagizi et al., 2020), impaired physical performance, mental health disturbances (Deslandes et al., 2009; Mahindru et al., 2023), type 2 diabetes mellitus (Tessier et al., 2000), hypertension (Borjesson et al., 2016), and chronic inflammation (Geffken et al., 2001). Despite this evidence, assessing the adequacy of physical activity remains challenging because external workload or performance does not necessarily reflect the magnitude of internal physiological stress. As a result, physical activity is frequently evaluated indirectly through subjective measures of perceived exertion or well-being (Steptoe et al., 2015; Stults-Kolehmainen, 2023) or by secondary physiological markers (Gutin and Owens, 2011; Xu et al., 2015), which may not fully capture the complexity and inter-individual variability of stress responses. This underscores the need for internal biomarkers that more precisely reflect individual stress and early recovery dynamics.

**Figure 1:**
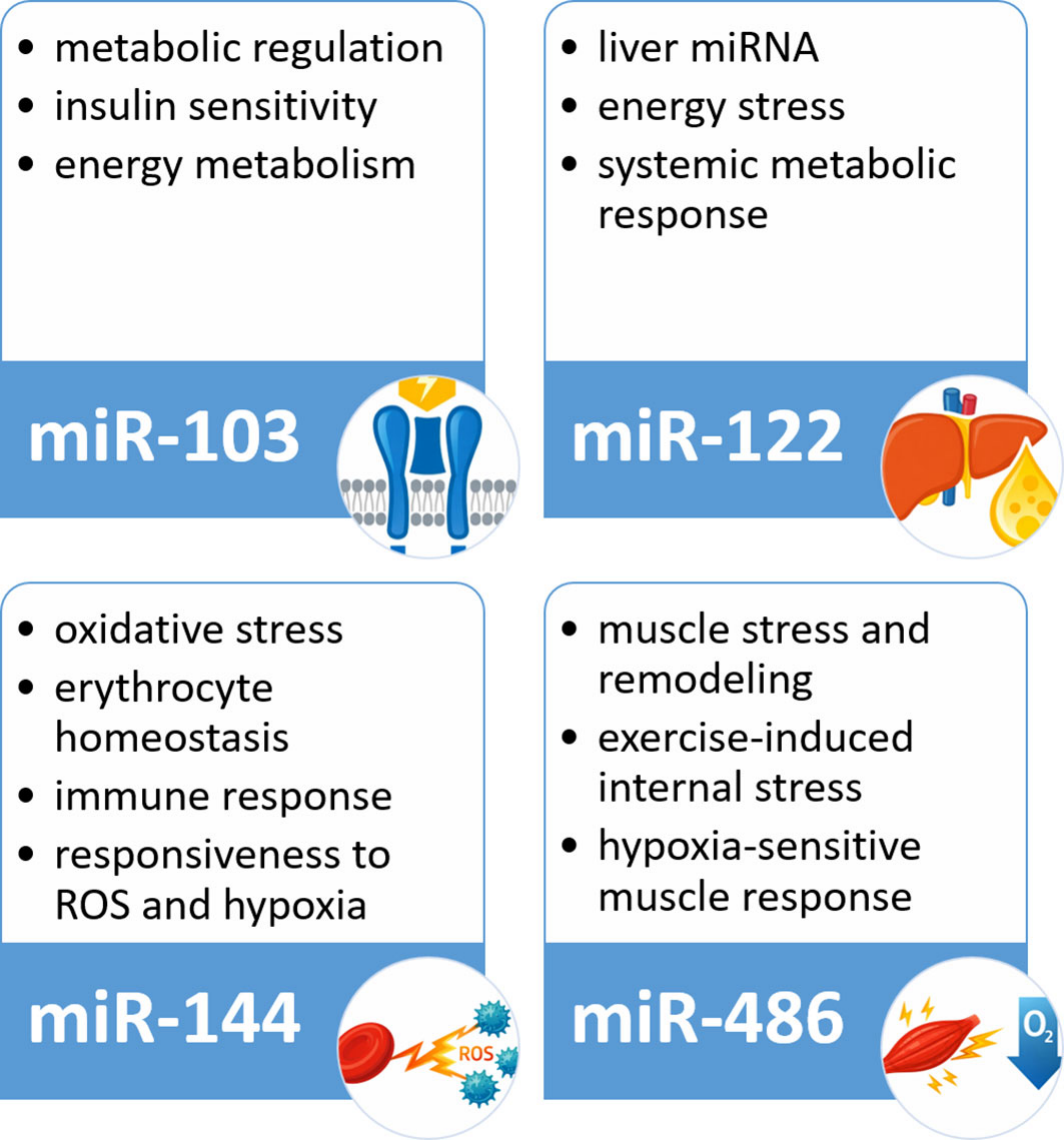
Physiological functions of selected circulating miRNAs involved in this study. Selected miRNAs represent distinct but partially overlapping regulatory axes involved in metabolic regulation (miR-103, miR-122), oxidative and redox balance (miR-144), and skeletal muscle-related internal stress responses (miR-486) during acute exercise and early recovery.

Here, we focused on a targeted panel of circulating miRNAs selected *a priori* to represent distinct yet partially overlapping axes relevant to acute exercise and early recovery: metabolic regulation (miR-103, miR-122), oxidative/redox regulation (miR-144), and muscle-related stress signaling (miR-486) (**Figure 1**). miR-103 (often co-expressed with miR-107) has been implicated in glucose metabolism and insulin sensitivity: inhibition of miR-103/107 enhances insulin receptor stability and improves glucose tolerance, whereas overexpression disrupts metabolic control (Trajkovski et al., 2011). miR-103 has also been linked to inflammatory regulation in adipose tissue via mitophagy-related mechanisms (Zhang et al., 2019), supporting its role in adaptive responses rather than disease itself (Trajkovski et al., 2011).

miR-122 is a liver-enriched miRNA and key regulator of lipid and cholesterol metabolism (Lagos-Quintana et al., 2002; Sacco and Adeli, 2012); its inhibition reduces circulating cholesterol, enhances hepatic fatty acid oxidation, and activates AMP-activated protein kinase (Esau et al., 2006), making it relevant to systemic energy balance during and after exercise. miR-144 and miR-486 have been investigated primarily in cellular stress responses and tissue adaptation: miR-144 is associated with oxidative stress compensation and cholesterol homeostasis (Sangokoya et al., 2010; Sharma et al., 2021), whereas miR-486 relates to skeletal muscle processes, stress signaling, and tissue protection (Al Azzouny et al., 2021; Vinas et al., 2021). Although these miRNAs are often described as disease biomarkers, their physiological functions suggest involvement in adaptation to acute physical stress and subsequent recovery.

Cortisol remains one of the most widely used stress biomarkers in exercise physiology. Its secretion follows a pronounced circadian rhythm, and the free fraction readily diffuses into saliva, where it reflects circulating free cortisol levels; salivary cortisol is therefore recommended as a practical and non-invasive indicator of training and exercise-induced stress (Cevada et al., 2014). Notably, miRNAs have been reported to modulate glucocorticoid biosynthesis and cortisol metabolism (Clayton et al., 2018), indicating potential interactions between hormonal and post-transcriptional regulatory pathways during stress adaptation. However, despite extensive research on exercise-induced changes in circulating miRNAs, findings remain highly heterogeneous, likely reflecting differences in exercise protocols, participant characteristics, and sampling time points, as well as the context-dependent nature of miRNA regulation. Importantly, many studies rely on simple pre- versus post-exercise comparisons and emphasize changes in individual miRNAs, treating them as isolated biomarkers rather than as potentially coordinated regulatory signals. In the present work, we therefore adopt an exploratory coordination-based perspective, operationalized as phase-specific changes in correlation structure among miRNA responses, to complement mean-level comparisons and to better reflect context-dependent regulation across acute exercise and early recovery.

Accordingly, the present study aimed to characterize phase-specific circulating miRNA responses to acute maximal-effort exercise and early recovery, with emphasis on miRNA coordination patterns and their associations with internal and external indicators of exercise load; salivary cortisol was included as a reference marker of systemic stress. Specifically, we investigated (i) acute miRNA and cortisol responses immediately post-exercise, (ii) early recovery dynamics relative to baseline and post-exercise levels, and (iii) associations between molecular responses and internal physiological stress operationalized by exercise-induced tissue oxygen desaturation (ΔStO_2_) and salivary cortisol, in contrast to external load described by achieved power output and habitual training volume. We hypothesized that:

H1: internal physiological stress and recovery are associated with phase-specific circulating miRNA responses;
H2: the magnitude of muscle oxygen desaturation (ΔStO_2_) during acute exercise is associated with molecular (miR-486) and hormonal (cortisol) stress responses; and
H3: circulating miRNA responses are organized into phase-specific coordinated modules rather than forming a single signature.

These hypotheses were defined *a priori* to test an internal-load framework. Mechanistic interpretation of observed associations is therefore presented as hypothesis-generating and is developed in the Discussion.

## 2 Materials and methods

**Figure 2:**
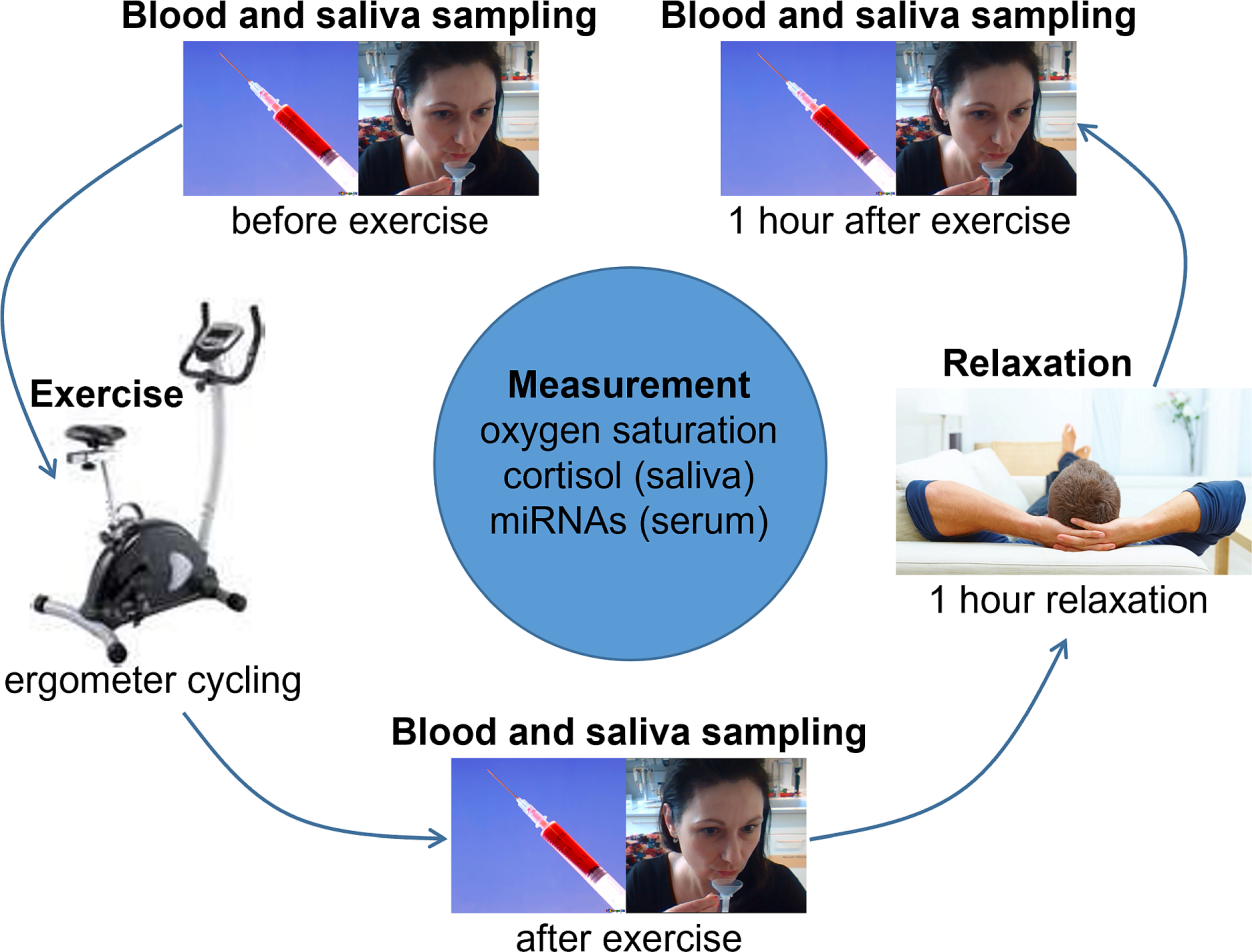
Overview of the experimental protocol.

The experiment (depicted in **Figure 2**) was performed over two days for several hours, with 11 young men (19–23 years) probands in total. Probands were instructed to keep alcohol-free, engage in low physical exercise, eat healthy food, and take no supplements for three days before the examination. Their saliva (1 mL) and blood (3 mL) were sampled three times: (1) before the exercise, (2) immediately after the exercise, and (3) 1 hour after the end of the exercise (resting time). During the experiment, the probands were allowed to drink table water only. Before the performance, they were asked to provide short but intensive (their maximum, estimated from body weight) exercise (cycling in an ergometer, allowing them to control the effort). We measured the oxygen saturation (StO_2_) of peripheral tissues, cortisol level in saliva samples, and four miRNAs (103, 122, 144, and 486) in serum samples, followed by a general questionnaire focused on the description of the usual amount of physical exercise per week (see SI).

The protocol comprised baseline assessment, maximal-effort cycling exercise, and 1 h recovery. Blood and saliva were sampled at baseline, immediately post-exercise, and after recovery. Tissue oxygen saturation was assessed non-invasively during exercise, while salivary cortisol and circulating miRNAs were determined from collected samples.

### 2.1 Exercise protocol

The exercise protocol consisted of a short-duration, maximal-effort cycling task performed on an ergometer. Participants were instructed to reach their individual maximal effort while being allowed to self-regulate cadence and resistance; the protocol was designed to elicit a pronounced acute physiological stress response rather than to standardize external workload across participants. Heart rate was monitored continuously using a chest-strap system. Exhaustion was operationalized using objective termination criteria selected for this age category: the test was terminated when participants reached a peak heart rate ≥192 bpm (consistent with near-maximal effort in young adults) and were unable to maintain a cadence of at least 60 rpm despite standardized verbal encouragement. Peak power output and body-mass–normalized peak power output were recorded and used as descriptors of external exercise load.

### 2.2 miRNA analysis

Blood samples were centrifuged immediately after sampling (2200–2500 *rpm for* 10 minutes) to obtain serum, which was subsequently stored at -80 °C until analysis. Circulating miRNA levels were determined by quantitative real-time PCR (qRT-PCR) using a previously published protocol (Forero et al., 2019).

Total miRNA was extracted from 250 µL of serum using the CatchGene Catch-miRNA Serum/Plasma kit (BioVendor, Czech Republic), according to the manufacturer’s instructions. Reverse transcription was performed using the TaqMan™ MicroRNA Reverse Transcription Kit (Thermo Fisher Scientific, USA). Individual miRNAs were quantified using specific TaqMan Advanced miRNA Assays in combination with TaqMan Advanced miRNA Assay Master Mix (Thermo Fisher Scientific). Samples were analyzed by qRT-PCR according to the manufacturer’s instructions. All samples were analyzed in triplicate using the RotorGene RG-3000A (Corbett Research, United Kingdom). Threshold cycles (C_t_) were determined using RotorGene 6 software (Corbett Research). To minimize technical variability (sample handling, RNA yield, and PCR inhibition), data were normalized to an endogenous reference miRNA. Based on assay optimization in the present sample set, miR-16 showed the most stable C_t_ values across participants and time points and was therefore selected as the reference miRNA. Because erythrocyte-related miRNAs can be influenced by hemolysis, this potential confound cannot be excluded by physiological monitoring and is addressed in the study limitations. Relative miRNA expression levels were calculated using the comparative C_t_ method. ΔC_t_ values were calculated as the difference between the C_t_ of the target miRNA (ΔC_t_ = C_t__target − C_t__reference), and relative expression was calculated as R = 2^(−ΔC_t_). Changes in miRNA expression between time points were expressed as fold changes. Fold-change values were subsequently log_2_-transformed (log_2_FC) to stabilize variance, reduce inter-individual variability, and enable comparison across biomarkers.

### 2.3 Oxygen saturation determination

The percentage of hemoglobin binding sites in the tissue microvasculature occupied by oxygen is defined as tissue oxygen saturation (StO_2_). During exercise, the elevated oxygen demand of working muscle can decrease StO_2_, and oxygen uptake shows an inverse relationship with tissue oxygenation (Koppo et al., 2002; Krustrup et al., 2003). To avoid invasive monitoring during exercise, near-infrared spectroscopy (NIRS) was used to assess tissue oxygenation noninvasively. NIRS measurements were obtained from the *triceps brachii* (lateral head) using an Oxiplex TS device (ISS Inc., Champaign, IL, USA; sampling frequency 1 Hz). The probe was placed on the muscle belly according to SENIAM guidelines while the participant was seated on the cycle ergometer. In addition to StO_2_, the device provided hemoglobin-derived signals, including total hemoglobin concentration (TotHbConc), oxygenated hemoglobin concentration (OxyHbConc), and deoxygenated hemoglobin concentration (DeOxyHbConc). For the present analyses, values were extracted at baseline (1-min average before pedaling), during the 5th minute of exercise, and during the last minute of the exercise bout. Internal physiological stress exposure was summarized *a priori* as ΔStO_2_ (baseline to end-exercise difference) to provide a single interpretable metric of maximal deoxygenation during this short maximal-effort bout; more detailed time-series descriptors were not analyzed in this pilot study and are an important target for future work.

### 2.4 Cortisol determination

We have chosen the period for the testing typical for the basic cortisol levels (from 10 a.m. to 3 p.m.). The saliva samples were kept refrigerated and analyzed 16 hours later. The analysis was performed using the direct colorimetric enzymatic immunoassay using the salivary cortisol ELISA kit (DCM020-11; DiaMetra, Segrate, Italy). The color intensity was inversely proportional to the cortisol concentration in the sample and was measured using the HiPo MPP-96 Microplate photometer (BioSan, Latvia).

#### 2.4.1 Fold change and log_2_ transformation

Relative changes in circulating miRNA expression levels and cortisol concentrations were quantified as fold change (FC) between paired time points. Fold change was defined as the ratio of normalized values at two time points:


$$\text{FC}=\frac{X_{2}}{X_{1}}$$

where $X_{1}$ and $X_{2}$ represent the measured value (normalized miRNA expression or cortisol concentration) at the respective time points.

To stabilize variance, reduce right-skewness, and obtain symmetric distributions around zero, FC values were log_2_-transformed according to:


$${\text{log}}_{2}\text{FC}={\text{log}}_{2}(\frac{X_{2}}{X_{1}})$$

Positive log_2_FC values indicate up-regulation (increase relative to the reference time point), whereas negative values indicate down-regulation. Relative changes in circulating miRNA expression levels and salivary cortisol concentrations were expressed as fold change (FC) between paired time points and log_2_-transformed (log_2_FC) to stabilize variance and obtain symmetric distributions around zero. Positive log_2_FC values indicate an increase relative to the reference time point, whereas negative values indicate a decrease. Log_2_FC was calculated for three predefined contrasts: immediately after exercise vs. baseline (after/before), recovery vs. post-exercise (rest/after), and recovery vs. baseline (rest/before).

### 2.5 Statistical analysis

All statistical analyses focused on relative changes and phase-specific responses to acute exercise. Given the limited sample size for serum miRNA analyses and the anticipated inter-individual variability in molecular and physiological responses, a non-parametric statistical approach was applied throughout.

For miRNAs, relative expression values (2^−ΔC_t_) were used to calculate fold changes between time points, which were subsequently log_2_-transformed (log_2_FC) for statistical analysis. Three predefined contrasts were analyzed: acute response to exercise (after/before), recovery relative to baseline (rest/before), and active recovery from post-exercise levels (rest/after). Log_2_ transformation was applied to reduce inter-individual variability and to enable direct comparison across biomarkers with different absolute concentration ranges.

Salivary cortisol concentrations were analyzed analogously, using log_2_-transformed fold changes between corresponding time points. Tissue oxygen saturation (StO_2_) was analyzed as an absolute change (ΔStO_2_), calculated as the difference between baseline and end-exercise values, reflecting internal physiological stress during exercise. Exercise performance variables (absolute power output, relative power output expressed as W·kg^-1^, and weekly training volume) were analyzed in their original units.

Descriptive statistics are presented as medians and interquartile ranges unless stated otherwise. Paired comparisons of biomarker levels across time points were performed using the Wilcoxon signed-rank test. Associations between continuous variables were assessed using Spearman’s rank correlation coefficient (ρ), which was chosen to quantify monotonic relationships independent of data distribution.

Correlation analyses were conducted to examine relationships between miRNA responses, cortisol changes, tissue oxygen saturation, exercise performance, and training volume. Correlation matrices were constructed separately for each phase (after/before, rest/before, rest/after) to explore phase-dependent coordination or dissociation of molecular responses. Given the number of correlation tests across biomarkers and phases, correlation analyses were treated as exploratory and hypothesis-generating. Accordingly, nominal p-values are reported for transparency and interpreted cautiously in the context of effect size, consistency across phases, and the study limitations.

All statistical tests were two-tailed, and the level of statistical significance was set *a priori* at α = 0.05. Given the exploratory nature of the study and the limited sample size, emphasis was placed on the magnitude and direction of effects rather than sole reliance on p-values.

Statistical analyses were performed using IBM SPSS Statistics (version 24; IBM, USA) and Origin (OriginLab) for data processing and visualization.

## 3 Results

A total of 11 young men (19–23 years) were included in the study. Circulating miRNA (miR-103, miR-122, miR-144, miR-486) measured in serum, salivary cortisol, and tissue oxygen saturation (StO_2_) were available for all included participants. Self-reported habitual physical activity ranged from 0 to 18 h·week^-1^ (median 10 h·week^-1^; IQR 4–11.5). Peak cycling power output ranged from 110 to 240 W (mean ± SD: 186 ± 49 W), corresponding to 1.47–3.20 W·kg^-1^ (2.49 ± 0.63 W·kg^-1^). Body mass ranged from 62 to 86 kg (75.1 ± 6.4 kg). Individual-level performance and training descriptors are summarized in **Table 1**.

**Table 1:** Participant characteristics and exercise performance descriptors (n = 11).

Variable	Mean ± SD	Range	Median (IQR)
Age (years)	19–23	–	–
Body mass (kg)	75.1 ± 6.4	62–86	76 (74.5–78)
Peak power output (W)	186 ± 49	110–240	215 (140–220)
Peak power output (W·kg^-1^)	2.49 ± 0.63	1.47–3.20	2.82 (2.04–2.86)
Habitual exercise (h·week^-1^)	8.64 ± 5.64	0–18	10 (4–11.5)

Values are presented as mean ± SD (range) and median (IQR), where appropriate.

### 3.1 H1: Internal physiological stress and recovery are associated with phase-specific circulating miRNA responses

Acute molecular and hormonal responses to exercise were qualified as log_2_-transformed fold changes (after/before) and analyzed with the Wilcoxon signed-rank test (**Table 2**). No marker reached statistical significance, as all p-values exceeded 0.05. Among the investigated miRNAs, miR-122 demonstrated the largest acute response, with a median log_2_ fold change of −1.03 (IQR −2.12 to −0.60), corresponding to an approximately twofold decrease and a large effect size (r = 0.56), although the result did not reach statistical significance (p = 0.067). miR-103, miR-144 and miR-486 showed no significant immediate changes following exercise. Salivary cortisol showed a non-significant increase (median log_2_FC = 0.54; IQR −0.38 to 1.51; p = 0.206), indicating inter-individual variability in the stress response.

**Table 2:** Group-level acute responses of circulating miRNAs and cortisol to exercise.

Marker	Median log_2_FC	IQR	p-value	Effect size (r)
miR-103	−0.29	−0.53 to 0.11	0.278	0.35
miR-122	−1.03	−2.12 to −0.60	0.067	0.56
miR-144	−0.21	−0.66 to 0.10	0.206	0.40
miR-486	−0.20	−0.60 to 0.04	0.320	0.32
Cortisol	+0.54	−0.38 to 1.51	0.206	0.40

(Effect size r = |z|/√n; interpretation: ~0.1 small, ~0.3 middle, ≥0.5 high effect).

Recovery-related changes were quantified as log_2_-transformed fold changes between the recovery time point (1 h post-exercise) and baseline (rest/before) and analyzed using the Wilcoxon signed-rank test (**Table 3**). None of the investigated miRNAs (miR-103, miR-122, miR-144, miR-486) differed significantly from baseline; median log_2_FC values were close to zero and interquartile ranges included zero, indicating a return toward pre-exercise levels. In contrast, salivary cortisol remained significantly elevated (median log_2_FC = 0.69, IQR 0.15 to 1.56; p = 0.014), corresponding to an approximately 1.6-fold increase relative to baseline. Together, these findings indicate a dissociation between molecular and hormonal recovery kinetics, with circulating miRNAs normalizing rapidly while activation of the hormonal stress axis persists beyond the early recovery period.

**Table 3:** Group-level acute responses of circulating miRNAs and cortisol to exercise.

Marker	Median log_2_FC (rest/before)	IQR	p-value	Interpretation
miR-103	+0.28	−1.26 to +0.65	0.966	no lasting change
miR-122	−0.10	−2.16 to +0.79	0.700	return to baseline
miR-144	+0.06	−0.92 to +0.67	0.966	no lasting change
miR-486	+0.21	−0.40 to +0.64	0.831	no lasting change
Cortisol	+0.69	+0.15 to +1.56	0.014	significant persistent increase

(log_2_FC > 0 = higher value than baseline).

### 3.2 H2: The magnitude of muscle oxygen desaturation (ΔStO_2_) during acute exercise is associated with molecular (miR-486) and hormonal (cortisol) stress responses

To explore mechanistic links between physiological stress and molecular responses, associations between changes in tissue oxygen saturation (ΔStO_2_), miRNA expression and cortisol were assessed using Spearman correlation analysis. A significant positive correlation was observed between ΔStO_2_ and the acute change in miR-486 expression (ρ = 0.68, p = 0.021), indicating that greater peripheral desaturation during exercise was associated with a stronger molecular response. ΔStO_2_ was also positively correlated with the cortisol response (ρ = 0.61, p = 0.045), supporting its role as a marker of physiological stress. A trend toward correlation was observed between miR-486 and cortisol responses (ρ = 0.58, p = 0.061), suggesting coordinated regulation of molecular and hormonal stress pathways.

**Figure 3:**
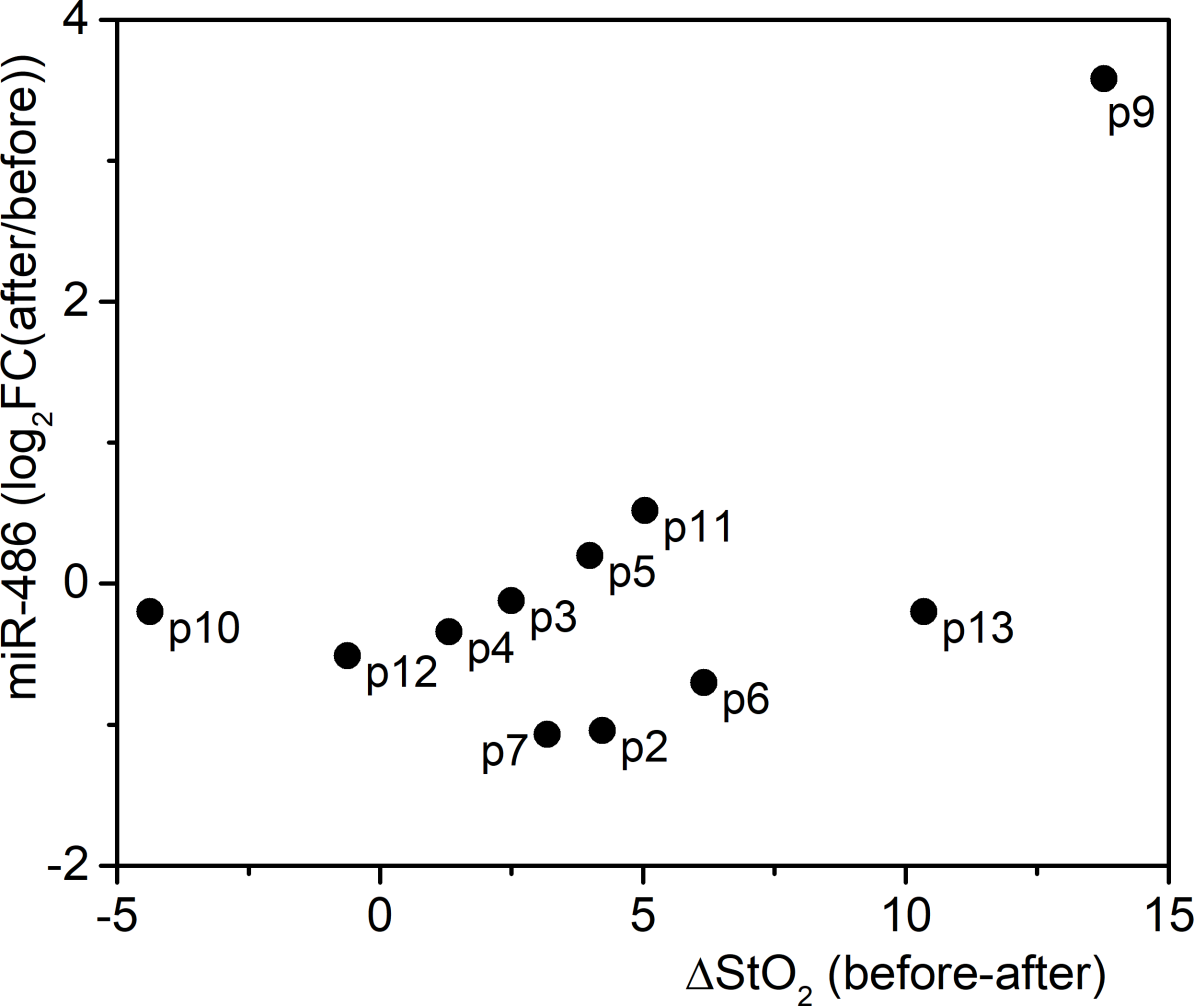
Association between peripheral tissue oxygen desaturation and miR-486 response.

**Figure 4:**
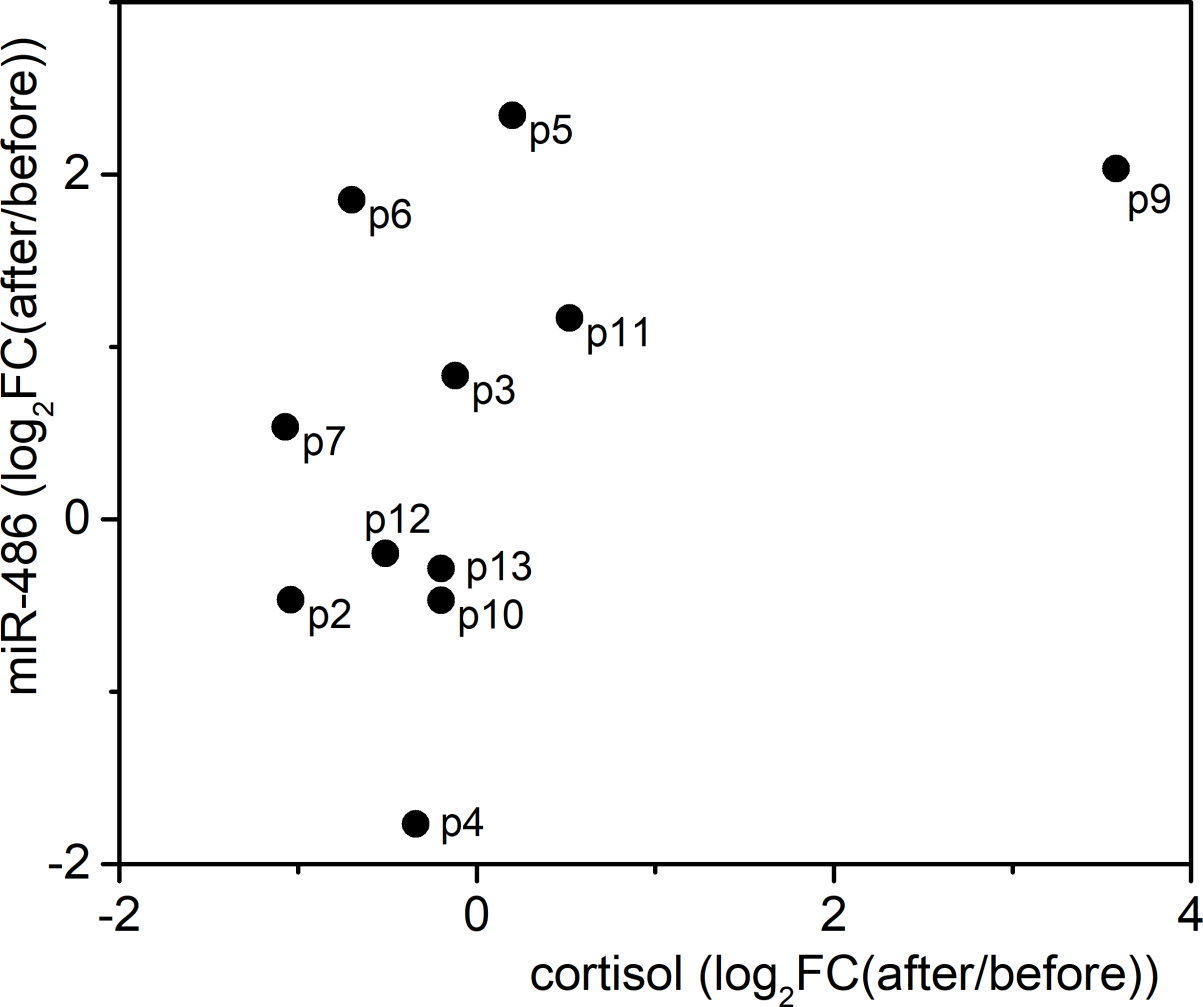
Association between cortisol and miR-486 response.

The relationship between the absolute change in tissue oxygen saturation (ΔStO_2_ = before − after exercise) and cortisol and the acute miR-486 response expressed as log_2_-transformed fold change (after/before) is shown for individual participants (n = 11) (**Figures 3**, **4**). Each point represents one subject. Spearman’s rank correlation coefficient (ρ) and the corresponding p-value are indicated. The dashed line represents a linear trend for visual guidance only.

#### 3.2.1 Training status and inter-individual variability in molecular responses

Training status, expressed as weekly training volume (hours per week), was explored as a potential modulator of exercise-induced responses. A moderate negative association was observed between training volume and the acute miR-486 response (Spearman ρ = −0.51, p = 0.11), suggesting smaller miR-486 responses at higher training volumes. Training volume also showed a negative trend with peripheral desaturation (ΔStO_2_; ρ = −0.39, p = 0.24), whereas no associations were found between training volume and miR-103, miR-122, or cortisol responses. Although these relationships did not reach statistical significance, their direction and magnitude are reported as exploratory observations in the context of the limited sample size (**Table 4**). Accordingly, these findings should be interpreted as hypothesis-generating rather than evidence of a training-related attenuation of molecular or physiological stress responses.

**Table 4:** Spearman correlations between weekly training volume and acute molecular, hormonal, and oxygenation responses.

Outcome	Spearman ρ	p-value	Interpretation
miR-486 log_2_FC (after/before)	−0.51	0.110	moderately strong negative relationship
miR-122 log_2_FC (after/before)	+0.05	0.894	no relationship
miR-103 log_2_FC (after/before)	−0.15	0.650	no relationship
Cortisol log_2_FC (after/before)	+0.02	0.958	no relationship
ΔStO_2_ (baseline − end-exercise)	−0.39	0.235	weaker negative relationship

Exercise performance was further explored as a potential determinant of molecular and physiological responses using Spearman’s rank correlation analysis. To account for differences in body mass, achieved power was additionally normalized to body weight (W·kg^-1^). Neither absolute achieved power nor power normalized to body mass showed significant associations with changes in tissue oxygen saturation (ΔStO_2_), miR-486 log_2_ fold change, or salivary cortisol responses (all p > 0.05; **Supplementary Table SI1**).

### 3.3 H3: Relationships between miRNAs – searching for miRNA signature

#### 3.3.1 miRNA–miRNA coordination during acute exercise

Associations among acute miRNA responses were explored using Spearman correlation analysis (**Supplementary Tables SI2, SI3**) of log_2_-transformed fold changes (after/before). The observed positive correlation between miR-122 and miR-486 responses (ρ = 0.63, p = 0.039) suggests coordinated regulation of metabolically and muscle-related stress pathways in response to acute exercise. Individuals exhibiting a stronger suppression of miR-122 also showed a more pronounced miR-486 response, consistent with coordinated behavior between these miRNAs during the acute phase.

In contrast, the inverse trend between miR-103 and miR-486 (ρ = −0.48), although not statistically significant (p = 0.13), may indicate a potentially modulatory relationship between metabolic regulatory processes and muscle-related stress signaling.

miR-144 did not exhibit meaningful correlations with any of the other investigated miRNAs, indicating that its acute response to exercise is largely independent of the metabolic and muscle-related miRNA axes.

Acute exercise elicits coordinated but non-uniform miRNA responses, revealing parallel regulatory axes rather than a single circulating miRNA signature. The miR-122–miR-486 association highlights phase-specific coordination between metabolic- and muscle-related miRNA responses during acute exercise.

#### 3.3.2 miRNA–miRNA coordination during recovery (phase-dependent reorganization of miRNA coordination during recovery)

During the recovery phase (log_2_ rest/before), the pattern of miRNA associations differed markedly from the acute post-exercise response (**Supplementary Tables SI4, SI5**). In contrast to the selective coordination seen immediately after exercise, recovery was characterized by the emergence of broader, cross-domain miRNA synchronization.

Moderate positive associations were observed between miR-103 and miR-144 (ρ = 0.54, p = 0.089) and between miR-103 and miR-486 (ρ = 0.57, p = 0.066), indicating coordinated regulation across metabolic, oxidative, and muscle-related pathways during the return toward baseline. Although these relationships did not reach conventional statistical significance, their consistency and effect sizes are reported as exploratory and hypothesis-generating in the context of the limited sample size.

Notably, miR-122 exhibited weak or inverse associations with the remaining miRNAs during recovery, including a moderate negative trend with miR-486 (ρ = −0.43). This contrasts with its coordinated behavior during the acute phase and suggests that liver-related metabolic regulation may follow a distinct temporal trajectory, becoming less coupled with the correlation patterns observed among muscle- and stress-related miRNAs during recovery.

Overall, the results indicate phase-specific changes in miRNA correlation structure: acute exercise showed selective associations, whereas early recovery displayed broader, exploratory cross-domain coordination patterns. While the acute phase primarily identified miRNAs responsive to exercise-induced stress, the recovery phase revealed coordinated regulation among miRNAs involved in metabolic, oxidative, and muscle-related pathways.

#### 3.3.3 Active recovery dynamics: miRNA coordination during post-exercise recovery (miRNA–miRNA coordination during active recovery (log_2_(rest/after)

During the active recovery phase, assessed as log_2_-transformed fold changes between the post-exercise and one-hour recovery time points (rest/after), a distinct and highly structured pattern of miRNA coordination emerged (**Supplementary Tables SI6, SI7**). A strong positive correlation was observed between miR-103 and miR-144 (ρ = 0.86, p = 0.0006), representing the most significant association detected across all analyzed phases and contrasts in the study. This finding indicates a tightly synchronized regulation of metabolically and oxidatively related miRNAs during the transition from acute exercise stress toward recovery.

In contrast, miR-486 exhibited a divergent recovery behavior, characterized by a moderate inverse association with miR-144 (ρ = −0.47, p = 0.14) and no meaningful relationship with miR-103. This suggests that miR-486 follows a distinct recovery trajectory, potentially reflecting differences in muscle-related or stress-responsive regulation compared with metabolico-oxidative pathways.

miR-122 showed no significant association with miR-103 or miR-144 during active recovery, remaining largely independent of the coordinated metabolic–oxidative axis. A trend toward a positive association with miR-486 (ρ = 0.52, p = 0.10) was observed, indicating a potentially shared but temporally delayed recovery pattern distinct from the tightly coupled miR-103–miR-144 response.

### 3.4 Integrated phase-specific miRNA response to acute exercise and recovery

**Figure 5:**
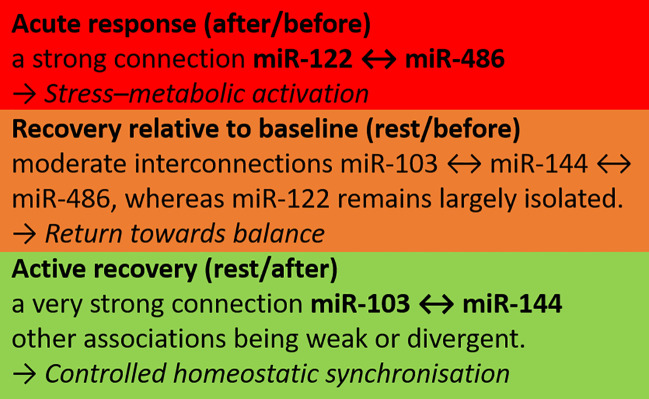
Phase-specific coordination of circulating miRNA responses to maximal-effort exercise. Schematic summary of phase-dependent relationships between circulating miRNAs following maximal-effort exercise. Acute response (after/before) is characterized by a strong association between miR-122 and miR-486, reflecting stress-related metabolic activation. Recovery relative to baseline (rest/before) shows moderate interconnections among miR-103, miR-144 and miR-486, while miR-122 remains largely independent, indicating a gradual return toward physiological balance. Active recovery (rest/after) is dominated by a very strong coupling between miR-103 and miR-144, with other associations being weak or divergent, suggesting a controlled and coordinated homeostatic synchronization during early recovery.

Taken together (**Figure 5**), the present results indicate a phase-dependent restructuring of circulating miRNA responses following maximal-effort exercise. The acute phase was dominated by stress-related and metabolically oriented miRNA interactions, particularly the miR-122 and miR-486 relationship, consistent with an immediate systemic response to physiological stress.

## 4 Discussion

### 4.1 Main findings and conceptual implications

The present study demonstrates that short maximal-effort exercise does not elicit a uniform or global circulating miRNA signature, but instead induces a structured, phase-specific reorganization of miRNA relationships across the acute response and early recovery. In this article, “coordination/network” terminology is used descriptively to refer to phase-specific changes in correlation structure among miRNA responses; formal network inference methods were not applied. This observation is consistent with accumulating evidence that circulating miRNAs act as context-dependent regulators rather than stable, single-response biomarkers of physiological stress or adaptation (Malm et al., 2019; Xu et al., 2015).

Rather than parallel changes in absolute miRNA levels, our data reveal distinct and temporally shifting patterns of coordination between individual miRNAs, suggesting the existence of multiple, partially independent regulatory axes governing the molecular response to exercise. Such behavior aligns with previous reports describing miRNA involvement in diverse metabolic, oxidative and stress-related pathways, including glucose and lipid metabolism (Esau et al., 2006; Trajkovski et al., 2011), oxidative stress compensation (Sangokoya et al., 2010), and muscle- and stress-associated signaling (Clayton et al., 2018).

In the acute phase, exercise-induced stress was primarily reflected by stress–metabolic coupling between miR-122 and miR-486, whereas other miRNAs exhibited weak or absent associations. This pattern is compatible with the established role of miR-122 as a regulator of systemic lipid and energy metabolism (Lagos-Quintana et al., 2002; Sacco and Adeli, 2012) and with evidence linking miR-486 to muscle-related stress and metabolic signaling pathways (Al Azzouny et al., 2021; Vinas et al., 2021).

During recovery relative to baseline, this acute pattern was replaced by a more distributed network of moderate interrelationships involving miR-103, miR-144 and miR-486, indicating a gradual return toward physiological balance. Notably, miR-103 has been implicated in the regulation of insulin sensitivity and metabolic flexibility (Improta-Caria et al., 2022; Trajkovski et al., 2011), while miR-144 is known to respond to oxidative and hypoxic stress (Sangokoya et al., 2010), supporting the notion that recovery engages coordinated metabolic–oxidative regulatory mechanisms.

Most prominently, the active recovery phase was characterized by a highly selective and tightly synchronized interaction between miR-103 and miR-144, representing the strongest association observed across all analyzed phases. This finding suggests that the transition from post-exercise stress to recovery is governed by controlled and phase-specific molecular coordination rather than uniform normalization of individual biomarkers.

Collectively, these findings indicate that acute exercise induces a structured, temporally ordered reorganization of circulating miRNA networks rather than uniform biomarker shifts. The observed pattern delineates a distinct metabolico–muscular stress axis (miR-122–miR-486), a modulatory metabolic component (miR-103), and an oxidative stress–responsive element (miR-144), each contributing differently across exercise and recovery phases.

Together, these findings challenge the concept of a single “exercise-responsive miRNA profile” and instead support a model of dynamic, phase-dependent regulatory reorganization. Such a framework is in line with current perspectives viewing exercise as a systems-level perturbation, in which internal physiological stress and recovery processes are orchestrated through temporally distinct molecular pathways rather than through isolated or static biomarker responses (Cevada et al., 2014; Steptoe et al., 2015; Stults-Kolehmainen, 2023).

### 4.2 Acute phase: stress–metabolic activation and internal physiological load

In the acute phase following maximal-effort exercise, molecular responses were characterized not by uniform changes in individual miRNA levels, but by a selective coordination between miR-122 and miR-486. This association suggests a coupling between systemic metabolic regulation and muscle-related stress signaling immediately after exercise. miR-122 is predominantly expressed in the liver and is a key regulator of lipid and energy metabolism (Esau et al., 2006; Lagos-Quintana et al., 2002), whereas miR-486 has been repeatedly linked to skeletal muscle physiology, insulin signaling and stress-related pathways (Al Azzouny et al., 2021; Vinas et al., 2021). Their coordinated behavior during the acute phase therefore likely reflects an integrated stress–metabolic activation rather than an isolated tissue-specific response.

Acute changes in miR-486 were associated with the magnitude of exercise-induced tissue deoxygenation (ΔStO_2_), whereas no meaningful relationships were observed with external performance metrics such as absolute power output (W) or body-mass–normalized power (W·kg^-1^). This dissociation highlights the primacy of internal physiological stress over externally defined workload in shaping acute molecular responses. Internal load markers are increasingly recognized as more accurate descriptors of individual strain and adaptation than external workload alone (Shahtahmassebi et al., 2019). Near-infrared spectroscopy indices of tissue oxygenation reflect local metabolic strain and oxygen utilization during high-intensity exercise (Koppo et al., 2002; Krustrup et al., 2003), and substantial inter-individual variability in microvascular perfusion and oxygen delivery/extraction may persist even at similar achieved power outputs; NIRS-derived tissue saturation metrics are designed to track this delivery–utilization balance *in situ* (Tuesta et al., 2022).

Mechanistically, the specificity of the miR-486 response to tissue deoxygenation (ΔStO_2_) is biologically plausible. miR-486 is a muscle-enriched “myomiR” involved in stress-responsive signaling pathways linking contractile activity to metabolic regulation. In muscle cells, miR-486 modulates PTEN and FOXO1, facilitating PI3K/Akt pathway activation, an axis central to acute regulation of glucose uptake, protein turnover, and cytoprotective responses under energetic strain (Small et al., 2010). Consistently, miR-486 is also implicated in hypoxia/ischemia-related contexts and may influence angiogenic/adaptive responses via PTEN/Akt signaling (Bao et al., 2024), aligning with the interpretation that larger exercise-induced deoxygenation captures a stronger “ischemia-like” component of local muscle stress than external workload metrics alone. In parallel, miR-122 is strongly liver-enriched and plays a role in systemic lipid and glucose metabolism (Bandiera et al., 2015; Willeit et al., 2017), and the coordinated behavior of miR-122 and miR-486 may therefore reflect an integrated liver–muscle metabolic response during acute maximal exertion.

### 4.3 Early recovery: molecular normalization and sustained HPA-axis activation

One hour after maximal-effort exercise, circulating miRNAs showed no consistent deviation from baseline at the group level, with median log_2_ fold changes close to zero and interquartile ranges spanning both directions. This pattern indicates rapid normalization of the analyzed miRNA signals during early recovery rather than a persistent post-exercise shift. In contrast, salivary cortisol remained elevated after one hour of rest, indicating sustained activation of the hypothalamic–pituitary–adrenal (HPA) axis beyond the time window in which miRNA levels largely returned toward baseline. Salivary cortisol is widely used as an index of circulating free cortisol and a practical marker of physiological stress and recovery dynamics (Cevada et al., 2014).

The divergence between molecular and hormonal kinetics suggests that endocrine and gene-regulatory responses may follow partially independent temporal trajectories during early recovery. Whereas circulating miRNAs reflect distributed regulatory signaling across tissues, cortisol kinetics depend on endocrine secretion, clearance, and feedback regulation within the HPA axis. miRNAs have also been implicated in glucocorticoid action and regulation, including pathways related to cortisol biosynthesis and metabolism, supporting the view that miRNA and cortisol systems can be linked yet temporally misaligned during recovery (Clayton et al., 2018). From a sport-science perspective, this dissociation reinforces the value of distinguishing rapid molecular “normalization” from slower endocrine recovery and aligns with evidence that cortisol recovery trajectories depend on exercise intensity and the magnitude of the physiological perturbation (Caplin et al., 2021).

Importantly, an apparent “return to baseline” in miRNA abundance should not be interpreted as an absence of regulation. In highly regulated systems, recovery may proceed through reorganization of interactions among regulatory elements rather than sustained directional changes in their absolute levels. From a network perspective, recovery can manifest as a shift from a perturbed coordination structure toward a more integrated and stable configuration without requiring persistent net changes in each component (Barabási et al., 2011). Accordingly, recovery vs baseline correlations were consistent with progressive reintegration across metabolic, oxidative, and muscle-related miRNA axes and motivated analysis of coordination during the transition from post-exercise to 1 h recovery (rest/after; Section 4.4).

### 4.4 Active recovery coordination: the miR-103–miR-144 regulatory axis

The most distinctive finding of the present study emerged during the active recovery phase, when molecular dynamics were evaluated relative to post-exercise levels (rest/after). In this phase, a very strong positive association between miR-103 and miR-144 was observed, representing the strongest miRNA–miRNA relationship detected across all analyzed conditions. Notably, this coupling was absent during the acute response and only weakly present when recovery was assessed relative to baseline, indicating that it is specific to the transition from post-exercise stress toward physiological stabilization rather than a simple return to pre-exercise levels.

From a biological perspective, the coordinated behavior of miR-103 and miR-144 suggests the engagement of complementary regulatory pathways governing metabolic and oxidative homeostasis during early recovery. miR-103 has been implicated in the regulation of insulin sensitivity, glucose utilization and metabolic flexibility (Improta-Caria et al., 2022; Trajkovski et al., 2011), whereas miR-144 has been associated with oxidative stress responses, erythrocyte function and redox regulation (Sangokoya et al., 2010; Sharma et al., 2021). Their tight synchronization during active recovery therefore points toward an integrated control mechanism in which metabolic recalibration and redox balance are jointly regulated as the organism exits the stress state induced by maximal-effort exercise.

Importantly, this highly selective coupling contrasts with the behavior of miR-486 and miR-122, which exhibited weaker, divergent or phase-independent associations during recovery. miR-486, despite its close link to muscle stress and exercise-induced perturbation, followed a distinct recovery trajectory, while miR-122 remained largely uncoupled from the metabolically and oxidatively regulated miRNAs, consistent with its predominantly hepatic origin and systemic metabolic role. Together, these findings indicate that recovery is not characterized by uniform molecular “cooling down”, but rather by selective and phase-specific coordination among particular regulatory axes.

Conceptually, the observed miR-103–miR-144 synchronization supports a model of recovery as an actively regulated process rather than a passive decay of stress signals. This view aligns with emerging systems-level perspectives in exercise physiology, which propose that recovery involves coordinated reorganization across metabolic, oxidative and regulatory networks, rather than simple reversal of acute exercise-induced changes (Laukkanen et al., 2021). In this context, circulating miRNAs may serve as sensitive indicators of how these regulatory layers are temporally aligned during the restoration of homeostasis.

The following mechanistic links are proposed as hypotheses for future testing and cannot be inferred from correlations alone. During active recovery, the emergence of a tightly synchronized miR-103–miR-144 coupling can be interpreted as a *regulated transition* from the immediate post-exercise perturbation toward stabilization, in which metabolic control and redox homeostasis become explicitly co-managed. Mechanistically, miR-103 (often discussed together with miR-107) is positioned within insulin-signaling control, where it regulates insulin sensitivity via targets such as caveolin-1 and thereby influences insulin receptor stability and downstream signaling; an axis directly relevant to early recovery processes such as glucose disposal and glycogen resynthesis (Trajkovski et al., 2011). In parallel, miR-144 is strongly implicated in oxidative-stress regulation through repression of NRF2, a central transcriptional regulator of antioxidant defense and glutathione recycling, and has also been described as hypoxia-responsive in cellular stress contexts (Gu et al., 2016; Sangokoya et al., 2010). The fact that this coupling becomes most apparent specifically in the *rest/after* phase supports the hypothesis that, once the acute “damage/strain” signals abate, the system shifts toward coordinated optimization of substrate handling and redox buffering, with active recovery (via maintained perfusion and oxygen flux) acting as a physiological context in which the linkage between insulin-metabolic regulation (miR-103) and antioxidant/hypoxia-related control (miR-144) becomes functionally meaningful and therefore more tightly coordinated at the circulating level.

### 4.5 Conceptual shift beyond the search for a single “exercise miRNA signature”

The present findings suggest that this inconsistency may not primarily reflect methodological noise, but rather a conceptual limitation of the “single-signature” approach (Amar et al., 2024). Our data indicate that exercise-induced miRNA responses are inherently phase-dependent and organized through shifting patterns of coordination rather than through uniform changes in individual miRNA abundance.

By analyzing miRNA dynamics across distinct phases, acute response, recovery relative to baseline, and active recovery, we demonstrate that the biological meaning of miRNA changes depends critically on temporal context. Associations that are absent during the acute phase may emerge during recovery, and vice versa, highlighting that circulating miRNAs participate in dynamic regulatory networks whose structure reorganizes over time. This phase-dependent network view aligns with systems biology perspectives, in which physiological perturbations are understood as transient reorganizations of interacting regulatory layers rather than as linear cause-effect responses of isolated markers (Mudge and Harrow, 2016; Ray-Jones and Spivakov, 2021).

Importantly, a network-based, phase-specific framing of circulating miRNAs provides a natural bridge to established sport-science concepts, particularly the distinction between external and internal load. In line with this framework, miRNA dynamics (notably those involving miR-486) aligned more closely with internal physiological strain indexed by ΔStO_2_ than with achieved power output, reinforcing the primacy of individual strain over nominal workload in training monitoring and adaptation research (Shahtahmassebi et al., 2019).

Taken together, the present study supports a shift from searching for universal exercise-responsive miRNA signatures toward analyzing how specific miRNAs interact within temporally defined regulatory networks. Such an approach may help reconcile discrepancies across the literature and better capture the complexity of individualized exercise responses. Accordingly, circulating miRNAs should be viewed not as static biomarkers, but as dynamic indicators of phase-specific coordination within the broader physiological system responding to and recovering from exercise.

### 4.6 Limitations and future directions

Several limitations should be considered when interpreting the findings of the present study. First, the relatively small sample size limits statistical power and precludes definitive conclusions regarding population-level effects. Although the repeated-measures design enabled within-subject comparisons, the results should be interpreted as exploratory and hypothesis-generating rather than definitive. Importantly, the primary aim of the study was not to identify small mean shifts in individual biomarkers, but to explore phase-specific coordination patterns and temporal shifts in correlation structure among circulating miRNAs; however, correlation estimates can be unstable in small samples, and all correlation-based findings should therefore be interpreted as exploratory and hypothesis-generating.

Second, the study focused four preselected miRNAs with established links to metabolic, oxidative and stress-related processes. While this targeted approach allowed for biologically interpretable analyses, it does not reflect the broader complexity of the circulating miRNA network. Future studies employing comprehensive miRNA profiling and network-based analyses may provide a more integrative view of exercise-induced regulatory dynamics.

Third, tissue oxygen saturation was used as an indicator of internal physiological stress, additional markers of metabolic strain, autonomic regulation or inflammatory responses were not assessed. Although NIRS was recorded continuously, the present analyses relied on summary indices (e.g., baseline-to-end ΔStO_2_), and time-series descriptors such as nadir values, area-under-the-curve, or time below baseline were not evaluated; these metrics may better capture nonlinear oxygenation dynamics during maximal exercise and should be examined in future work. Incorporating multimodal physiological measurements, including lactate kinetics, heart rate variability, and inflammatory markers, would strengthen future research aimed at developing comprehensive models of individualized exercise responses and recovery.

Finally, the findings are limited to young, healthy men performing short maximal-effort exercise. Whether similar phase-dependent miRNA coordination patterns occur in women, older individuals, trained athletes, or under different exercise modalities and intensities remains to be determined.

## 5 Conclusion

Short maximal-effort exercise did not produce a uniform change in individual circulating miRNAs, but was associated with phase-specific shifts in miRNA coordination patterns across the acute response and early recovery. The acute phase showed coordinated behavior between miR-122 and miR-486 alongside associations with internal physiological strain indexed by exercise-induced tissue deoxygenation (ΔStO_2_), whereas mean miRNA levels largely returned toward baseline at 1 h post-exercise while salivary cortisol remained elevated. During active recovery, a strong miR-103–miR-144 coupling emerged. Collectively, these exploratory observations support a phase-specific, coordination-based interpretation of circulating miRNAs in relation to internal load and early recovery and should be confirmed in larger, hypothesis-driven studies.

## 6 Perspectives

These pilot data motivate a shift from searching for universal exercise-responsive miRNA panels toward testing phase-specific, network-level hypotheses aligned with internal load and recovery physiology. Future studies should (i) expand sampling density (e.g., 15–30 min, 2–4 h, and 24 h post-exercise) to resolve distinct regulatory waves; (ii) include women and broader fitness/age strata to assess generalizability and potential sex- and training-status modulation; and (iii) integrate complementary internal-load readouts (e.g., lactate kinetics, HRV/autonomic indices, inflammatory markers) to map miRNA coordination onto multi-system stress dynamics. Methodologically, larger cohorts should enable pre-registered network models and appropriate control of multiple testing, while improved miRNA source attribution (e.g., EV-enriched vs protein-bound fractions and hemolysis checks) may clarify tissue contributions to phase-specific signatures. If confirmed, phase-dependent miRNA coordination patterns could support more individualized monitoring frameworks that distinguish external workload from internal physiological strain and recovery state.

## Data Availability

The datasets generated and analyzed during the current study are not publicly available but are available from the corresponding author upon reasonable request.
