# Supplementary material for: Phase-dependent reorganization of circulating miRNA networks following short maximal exercise
Source: Front Physiol. 2026 Jun 3;17:1852791. doi: 10.3389/fphys.2026.1852791 (PMC13271730; doi:10.3389/fphys.2026.1852791)
Supplement: Supplementary file 1 [file Table1.docx]

**Supplementary Information**

Phase-dependent reorganization of circulating miRNA networks following short maximal exercise

**SI1. Questionnaire**

Gender: M / F / other Age:

Body weight: Body height:

Estimated hours of sport activity per week:

Types of the sport activity:

**Table SI1.** Relationship of power normalized to weight (W/kg) or absolute power (W) to ΔStO_2_, miR‑486 log_2_FC and cortisol log_2_C Spearman correlation (n = 11)

| **Relationship** | **Spearman ρ** | **p-value** | **Interpretation** |
| --- | --- | --- | --- |
| W/kg × ΔStO_2_ | 0.02 | 0.96 | No relationship |
| W/kg × miR-486 log_2_FC | 0.02 | 0.96 | No relationship |
| W/kg × cortisol log_2_FC | −0.30 | 0.37 | Weak, non-significant |
| W × ΔStO_2_ | 0.08 | 0.81 | No relationship |
| W × miR-486 log_2_FC | 0.08 | 0.81 | No relationship |
| W × cortisol log_2_FC | −0.21 | 0.53 | Weak, non-significant |

**Table SI2.** miRNA–miRNA coordination during acute exercise: Spearman correlation matrix (ρ; log₂ fold-changes after/before) evaluating coordinated responses and potential multi-axis regulation rather than a single miRNA signature.

|  | **miR-103** | **miR-122** | **miR-144** | **miR-486** |
| --- | --- | --- | --- | --- |
| **miR-103** | 1.00 | −0.05 | +0.29 | −0.48 |
| **miR-122** | −0.05 | 1.00 | +0.25 | +0.63 |
| **miR-144** | +0.29 | +0.25 | 1.00 | −0.09 |
| **miR-486** | −0.48 | +0.63 | −0.09 | 1.00 |

**Table SI3.** Statistical significance of miRNA–miRNA coordination during acute exercise: p-value matrix corresponding to Spearman correlations (ρ) of log₂ fold-changes (after/before).

|  | **miR-103** | **miR-122** | **miR-144** | **miR-486** |
| --- | --- | --- | --- | --- |
| **miR-103** | – | 0.87 | 0.39 | 0.13 |
| **miR-122** | 0.87 | – | 0.45 | 0.039 |
| **miR-144** | 0.39 | 0.45 | – | 0.79 |
| **miR-486** | 0.13 | 0.039 | 0.79 | – |

**Table SI4.** miRNA–miRNA coordination during recovery: Spearman correlation matrix (ρ; log₂ fold-changes rest/before) evaluating coordinated responses and potential multi-axis regulation rather than a single miRNA signature.

|  | **miR-103** | **miR-122** | **miR-144** | **miR-486** |
| --- | --- | --- | --- | --- |
| **miR-103** | 1.00 | +0.10 | +0.54 | +0.57 |
| **miR-122** | +0.10 | 1.00 | +0.20 | −0.43 |
| **miR-144** | +0.54 | +0.20 | 1.00 | +0.34 |
| **miR-486** | +0.57 | −0.43 | +0.34 | 1.00 |

**Table SI5.** Statistical significance of miRNA–miRNA coordination during recovery: p-value matrix corresponding to Spearman correlations (ρ) of log_2_ fold-changes (rest/before).

|  | **miR-103** | **miR-122** | **miR-144** | **miR-486** |
| --- | --- | --- | --- | --- |
| **miR-103** | – | 0.77 | 0.089 | 0.066 |
| **miR-122** | 0.77 | – | 0.56 | 0.19 |
| **miR-144** | 0.089 | 0.56 | – | 0.31 |
| **miR-486** | 0.066 | 0.19 | 0.31 | – |

**Table SI6.** miRNA–miRNA coordination during active recovery: Spearman correlation matrix (ρ) of log₂ fold-changes between one-hour recovery and immediate post-exercise (log_2_(rest/after)), capturing post-exercise network reorganisation.

|  | **miR-103** | **miR-122** | **miR-144** | **miR-486** |
| --- | --- | --- | --- | --- |
| miR-103 | 1.00 | +0.15 | +0.86 | −0.25 |
| miR-122 | +0.15 | 1.00 | +0.07 | +0.52 |
| miR-144 | +0.86 | +0.07 | 1.00 | −0.47 |
| miR-486 | −0.25 | +0.52 | −0.47 | 1.00 |

**Table SI7.** Statistical significance of miRNA–miRNA coordination during active recovery: p-value matrix corresponding to Spearman correlations (ρ) of log_2_(rest/after) fold-changes.

|  | **miR-103** | **miR-122** | **miR-144** | **miR-486** |
| --- | --- | --- | --- | --- |
| miR-103 | – | 0.65 | 0.0006 | 0.45 |
| miR-122 | 0.65 | – | 0.83 | 0.10 |
| miR-144 | 0.0006 | 0.83 | – | 0.14 |
| miR-486 | 0.45 | 0.10 | 0.14 | – |
